# Supplementary material for: Wall shear stress and pressure patterns in aortic stenosis patients with and without aortic dilation captured by high-performance image-based computational fluid dynamics
Source: PLoS Comput Biol. 2023 Oct 18;19(10):e1011479. doi: 10.1371/journal.pcbi.1011479 (PMC10635572; doi:10.1371/journal.pcbi.1011479)
Supplement: S1 Appendix — (PDF) [file pcbi.1011479.s001.pdf]

S1 Appendix. Flow rate and pressure waveforms

The velocity waveforms are taken from continuous wave (CW) spectral echocardiography data (see Fig 1). All physiological waveforms are projected based on a heartbeat of 60bpm (beats per minute). For the idealized geometry case, a heart rate of 72bpm is used to fabricate the velocity and pressure waveform. All flow and pressure waveforms are shown in Figs. A and B and, while their corresponding velocity waveform can be obtained using the reference values given in Table A.

Table A: Flow rate and pressure waveform reference values

Case	U_{peak} (m/s)	R_{out} (mmHg/cm3/s)	C_{out} (cm3/mmHg)	$p_{systole}$ (mmHg)	$p_{diastole}$ mmHg
TAVI 0	2.7	0.898	2.066	113	80
TAVI 1	2.1	1.210	1.351	118	80
TAVI 2	2.2	1.083	1.900	109	80
Idealized	2.7	1.127	1.153	113	80
TAVI 0 Restored	0.9	0.898	2.066	123	80
TAVI 0 Restored+	1.8	0.6737	0.950	113	80

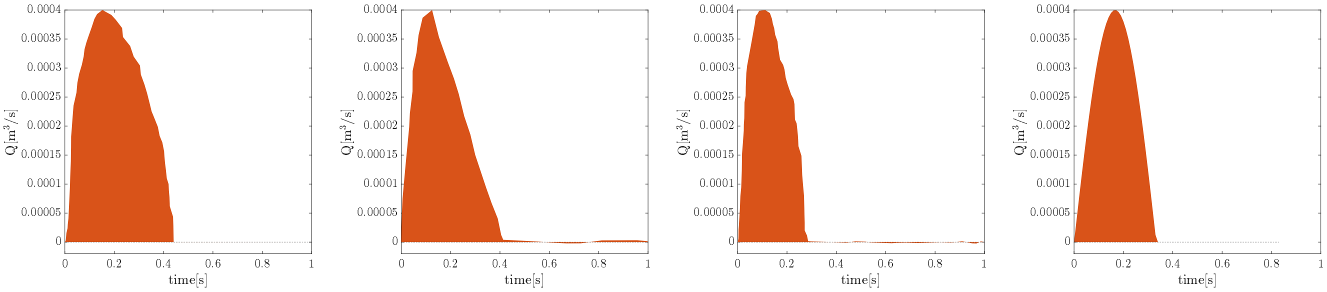

Fig. A: Flow rate waveforms from left to right, for cases TAVI0, TAVI1, TAVI2 and Idealized.

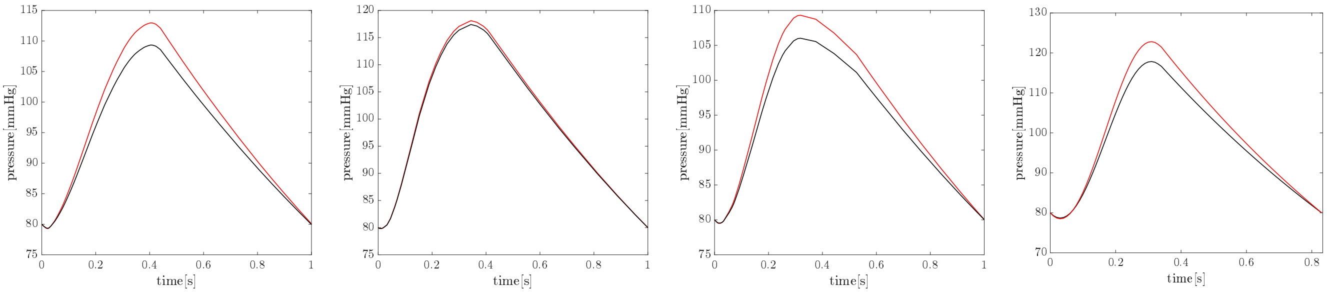

Fig. B: Pressure waveforms, from left to right, for cases TAVI0, TAVI1, TAVI2 and Idealized. Black curves show the waveform for the descending aorta outflow boundary, while the red curves show the corresponding waveforms at the inflow boundary.
