# Supplementary material for: Wall shear stress and pressure patterns in aortic stenosis patients with and without aortic dilation captured by high-performance image-based computational fluid dynamics
Source: PLoS Comput Biol. 2023 Oct 18;19(10):e1011479. doi: 10.1371/journal.pcbi.1011479 (PMC10635572; doi:10.1371/journal.pcbi.1011479)
Supplement: S2 Appendix — (PDF) [file pcbi.1011479.s002.pdf]

S2 Appendix. Verification of pressure discharge effect due to an upstream Brachiocephalic artery using a modified version of the Idealized model

We use a modified version of the Idealized model to show that a more proximal Brachiocephalic artery removes the focal zone of elevated WSS and pressure, but it leads to more aggressive flow disturbances. In the modified model geometry, the inlet of the Brachiocephalic artery is moved further upstream such that it coincides with the jet impingement zone (Fig. A).

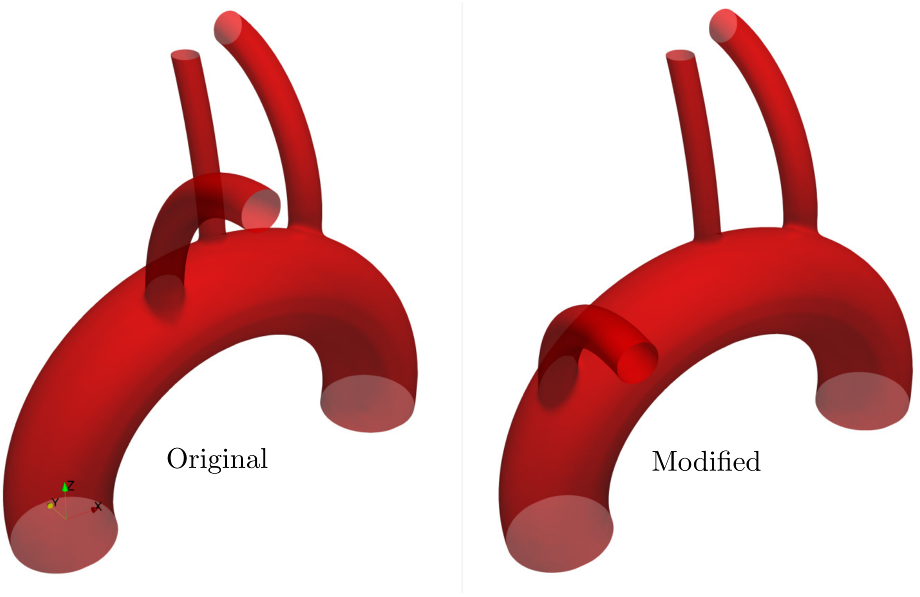

Fig. A: Geometry of the modified Idealized model (right) versus the original model (left). In the modified model, the Brachiocephalic artery is moved upstream where it branches from the location where the jet impingement occurs in the original model.

Fig. B shows that the pressure discharge occurs in the modified Idealized model, and removes the focal wall pressure zone from the aortic wall. The pressure signal shown at the bottom of this figure show that this case corresponds to larger flow disturbances compared to the original Idealized model (similar to TAVI1-2 in figure 10), and the pressure build up pattern can no longer be identified. This emphasizes the importance of spatial arrangement of the AS jet with respect to the streamwise location of the Brachiocephalic artery when assessing the aortic remodelling risk factors of aortic stenosis.

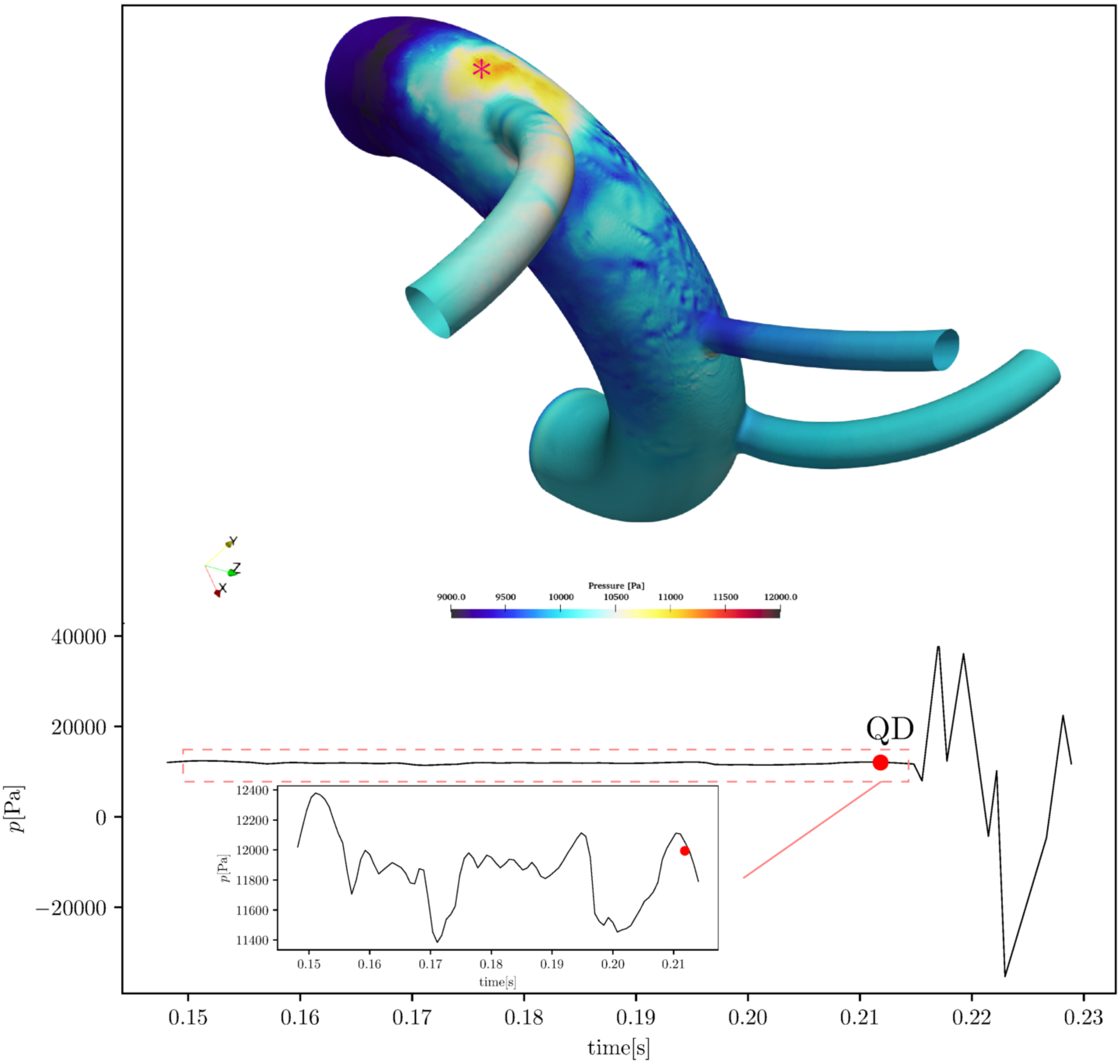

Fig. B: Wall pressure for the modified Idealized model. The pressure map on the top (taken at QD) shows that the Brachiocephalic artery (the artery facing the left side) has disturbed the pressure hotspot that was present in the original Idealized model. The temporal behaviour of pressure at a probe located close to the impingement zone is presented in the bottom of the figure. Pressure at this probe (marked by the asterisk on the pressure map) shows strong fluctuations instead of a pressure build up effect which was apparent in the original model (see Fig. 10). A part of signal (within the dashed red box) is magnified using the inset plot to show that no build up was present around QD (this instance is marked by the red bullet symbol in both main and inset plots).
